# Molecular Signaling and Nutritional Regulation in the Context of Poultry Feather Growth and Regeneration

**DOI:** 10.3389/fphys.2019.01609

**Published:** 2020-01-21

**Authors:** Meng-jie Chen, Wen-yan Xie, Shi-guang Jiang, Xiu-qi Wang, Hui-chao Yan, Chun-qi Gao

**Affiliations:** College of Animal Science, South China Agricultural University/Guangdong Provincial Key Laboratory of Animal Nutrition Control/Key Laboratory of Chicken Genetics, Breeding and Reproduction, Ministry of Agriculture, Guangzhou, China

**Keywords:** feather, feather follicle, stem cells, nutritional intervention, signaling regulation

## Abstract

The normal growth and regeneration of feathers is important for improving the welfare and economic value of poultry. Feather follicle stem cells are the basis for driving feather development and are regulated by various molecular signaling pathways in the feather follicle microenvironment. To date, the roles of the Wnt, Bone Morphogenetic Protein (BMP), Notch, and Sonic Hedgehog (SHH) signaling pathways in the regulation of feather growth and regeneration are among the best understood. While these pathways regulate feather morphogenesis in different stages, their dysregulation results in a low feather growth rate, poor quality of plumage, and depilation. Additionally, exogenous nutrient intervention can affect the feather follicle cycle, promote the formation of the feather shaft and feather branches, preventing plumage abnormalities. This review focuses on our understanding of the signaling pathways involved in the transcriptional control of feather morphogenesis and explores the impact of nutritional factors on feather growth and regeneration in poultry. This work may help to develop novel mechanisms by which follicle stem cells can be manipulated to produce superior plumage that enhances poultry carcass quality.

## Introduction

In modern commercial poultry production, the quantity and quality of feathering in both broilers and layers are gaining increased attention. The feather growth rate, quality and patterns of molting are important to the production of a high value poultry carcass (Guo, 2011). Poor feather growth not only affects the appearance of the organism but also decreases the uniformity of the carcass and feed efficiency. Thus, bad feather growth reduces the net profitability of poultry production (Lopez-Coello, 2003; Zeng et al., 2015). However, in the commercial production of poultry, plumage defects often occur, such as feather pecking, molting, and inadequate body coverage (Bajpai et al., 2016; Coton et al., 2019). Therefore, investigating the feather morphogenesis and development, signal transduction pathways, and effective nutrient interventions of poultry is of great economic significance.

## The Growth and Development of Poultry Feathers

### Feather Structure

**FIGURE 1:**
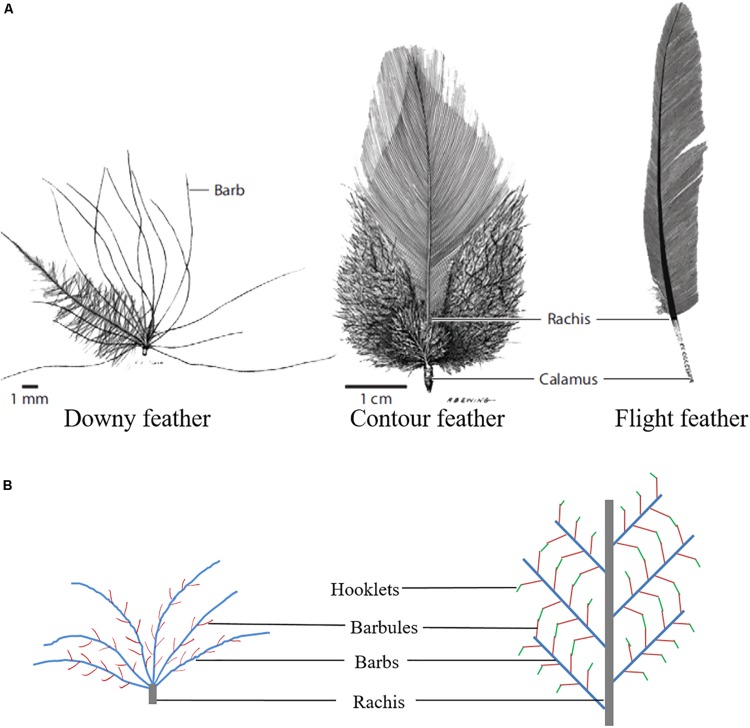
Feather branching morphogenesis. **(A)** Different types of chicken feathers (Lucas and Stettenheim, 1972). **(B)** Feather branching structure.

Feathers, which are unique epidermal structures originating from epidermal cells of the ectoderm, have a complex and fine structure. Feathers are not simply a flying tool, but also function as inulation, as well as aid in protection, swimming, temperature regulation, and a mode of communication (Clark et al., 2011; Prado et al., 2016). Poultry feathers have an extensive branching structure, and the development of feathers is the result of the proliferation and differentiation of feather follicle stem cells (Yue et al., 2005). Feather branching begins in the early stage of feather growth and consists of three levels: from rachis to barbs, from barbs to barbules, and from barbules to cilia or hooklets. These three levels of morphogenesis are combined to yield different types of feathers (Figure 1), which can be divided into symmetric down feathers, bilaterally symmetric contour feathers, and bilaterally asymmetric flight feathers (Chen et al., 2015). In addition, feather branching is strictly controlled by time and space. However, the molecular signal or cell fate determination mechanism involved in initiating feather branching remains an area to be further investigated.

### Feather Follicle Development

**FIGURE 2:**
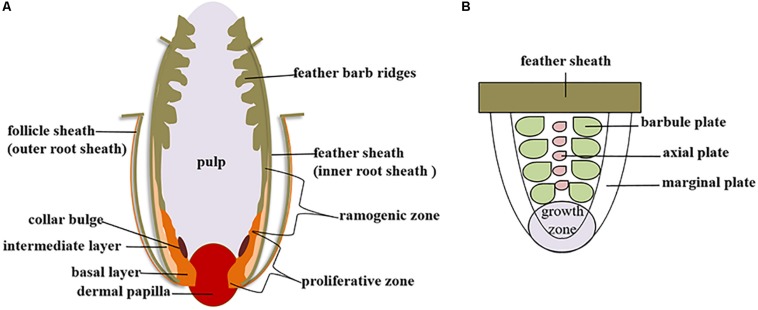
Poultry feather growth and development. **(A)** Diagram of the feather follicle structure. **(B)** Diagram of the feather barb ridge.

Feather follicles are formed by the interaction of dermal cells and epithelial cells, which is the basis for the growth and development of poultry feathers (Figure 2A). The dermis begins to form within in the developing plumage bearing skin due to rapidly proliferating mesenchymal cells on the 10th day of goose embryo development, and the process is completed by the 11th or 12th day. Then, dermal papillae are formed by the accumulation of columnar cells on the surface of the dermis, thus providing nutrients for feather growth (Wu et al., 2008). On the 13th or 14th days of goose embryo development, the dermal papilla grows thicker and forms a feather primordium together with the epithelial compartment. Then, the epidermis continues to bulge to form a feather bud (Jiang and Chuong, 1992), which further invaginates to form a primary feather follicle; secondary feather follicles are formed on the 18th day in the goose embryo (Wu et al., 2008).

For the chicken, feather buds are visible from the 5th to the 8th days of the embryonic stage, and feather buds began to gradually differentiate on the 9th day. Complete follicles and feathers are formed on the 17th day of hatching (Lucas and Stettenheim, 1972). In ducks, cell proliferation has been demonstrated to form feather buds at the epithelium on the 11th day of the embryonic stage. On the 15th day, the primary follicle forms, and the feather sheath fills in newly formed follicles. On the 20th day, the follicle and feather sheath are closely linked together to form a single layer, and feathers completely cover the body (Chen et al., 2012, 2017). Therefore, different types of poultry have different feather development patterns.

The growth of feathers is accompanied by the development of feather follicles. The dermal papilla grows upward to form a feather pulp, and endothelial cells invade and form capillary vessels, which transport nutrients within the dermal papilla to various parts of the feather (Alibardi, 2009). A proliferative zone exists at the bottom of the feather follicle, and a ramogenic zone lies above this area. In this zone, the rachidial and barb ridges are formed through epithelial-mesenchymal interactions. In a more distal position along the follicle, the barb ridge actively proliferates and differentiates to form the marginal plates, barbule plates and axial plates (Figure 2B). The marginal and axial plate cells later die, yielding the intervening space. Individual barbule plate cells undergo further cell shape changes to form cilia and hooklets. The barb ridges fuse proximally to form the rachidial ridge, which eventually becomes the rachis (Xu et al., 2007).

Feathers repetitively molt and regrow throughout the life of birds. Feathers can be regenerated naturally through molting or artificially by plucking. Chickens undergo more than 3–4 successions of feather growth and replacement to form adult plumage. The first feathers formed at the end of the embryonic stage are called downy feathers, the second generation is called juvenal feathers, the third is called youth feathers, and the fourth is the adult plumage. From this point, the feathers usually molt at regular intervals (Yu et al., 2004).

### Feather Follicle Regeneration

Because the feather follicle is a regenerating tissue, feathers can be produced cyclically throughout a bird’s life. Under normal circumstances, feather follicles can complete their own development through molecular signal transduction controlling cell proliferation and programmed cell death ensuring plumage coverage throughout life of the bird (Lin et al., 2013; Li et al., 2017). However, injury can also induce the feather follicle cycle to cover wounded skin regions.

Although the growth cycle of feather follicles differs among poultry species (Yue et al., 2005; Lin et al., 2013; Chen et al., 2017), it can be roughly divided into the following three stages: growth, resting and initiation (Lin et al., 2013). During the initiation phase, the feather primordium forms and then differentiates into a feather follicle under signal stimulation, and the feather begins to lengthen in the growth phase. During the resting phase, feather primordium differentiation is terminated through programmed cell death, halting feather growth and readying the structure to enter the next growth phase (Yu et al., 2004; Yue et al., 2005).

The presence of feather follicle bulge stem cells, confers the regenerate cycles of feather growth (Yue et al., 2005). The stem cells have strong proliferation, division and multidirectional differentiation potential, and can sense of changes in the follicle growth phase signals. These signals change from a resting state to an activated state or are activated in response to wounding, thereby the follicle bulge to participates in the repair of the damaged skin and promotes follicle morphogenesis or reconstruction (Yue et al., 2005). The periodic activation or resting state of feather follicle stem cells is the basic cause of the growth and degeneration of feather follicles. Therefore, increasing the activity and maintaining the normal state of feather follicle stem cells can restore feather loss. However, feather follicle stem cell activation and differentiation is controlled by the niche/microenvironment and the identification of mechanisms that can override that level of control will require further research.

## Molecular Signaling in Appendage/Feather Morphogenesis

The mammalian hair follicle and avian feather follicle are similar morphological structures and share many aspects of growth cycles, although they appear to have evolved independently. Due to dermal-epidermal cell interactions, feather follicles develop in the embryonic stage and undergo different cycles, including growth, resting and initiation phases. Mammalian hair follicles undergo four phases: anagen, catagen, telogen and exogen. Moreover, mature follicles also have a similar stem cell niche, inner root sheath (IRS), outer root sheath (ORS) and dermal papilla structures, but feather follicles have dermal pulp, while hairs do not (Chuong et al., 2000; Schlake, 2007; Alibardi, 2009). The feather follicle is ellipsoidal, while hair follicles are slender. The most important difference is that avian follicles produced branched to form different types of feathers. Mammalian hair development has been extensively studied in transgenic and knock-out mice and therefore the understanding of molecular signaling that controls the process is in some case more mature than that for avian feather development. Thus, in reviewing signaling pathways below, we will describe the conclusions for the hair follicle when work for the feather is incomplete.

**FIGURE 3:**
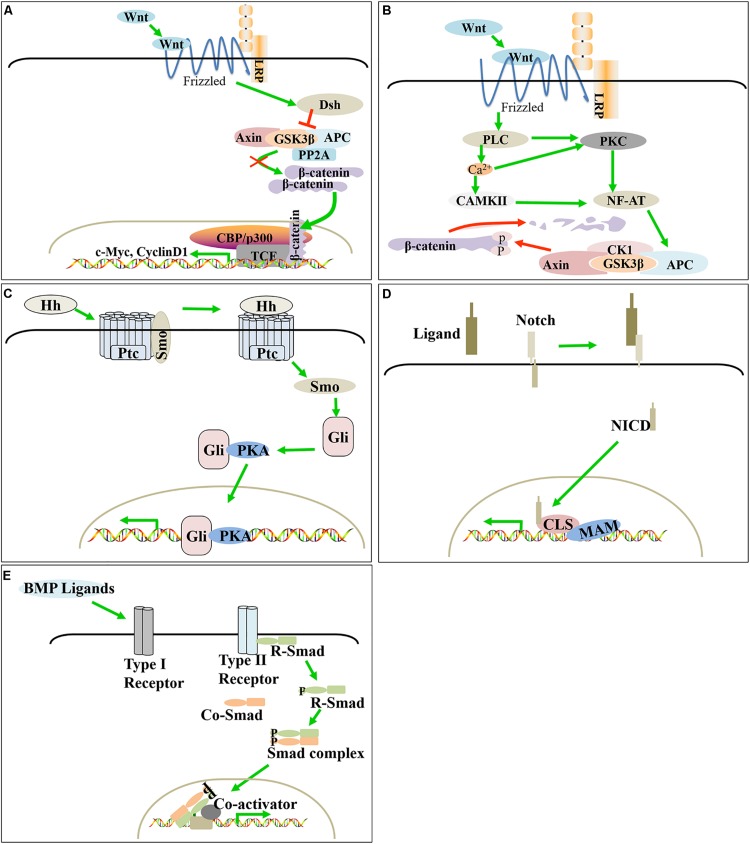
Molecular signaling in poultry feather follicle and feather development. Canonical Wnt/β-catenin, SHH, and Notch positively regulate feather follicle development, while BMP and the non-canonical Wnt signaling pathway negatively regulate feather follicle development. **(A)** Canonical Wnt/β-catenin signaling pathway. **(B)** Non-canonical Wnt signaling pathway. **(C)** SHH signaling pathway. **(D)** Notch signaling pathway. **(E)** BMP signaling pathway.

**FIGURE 4:**
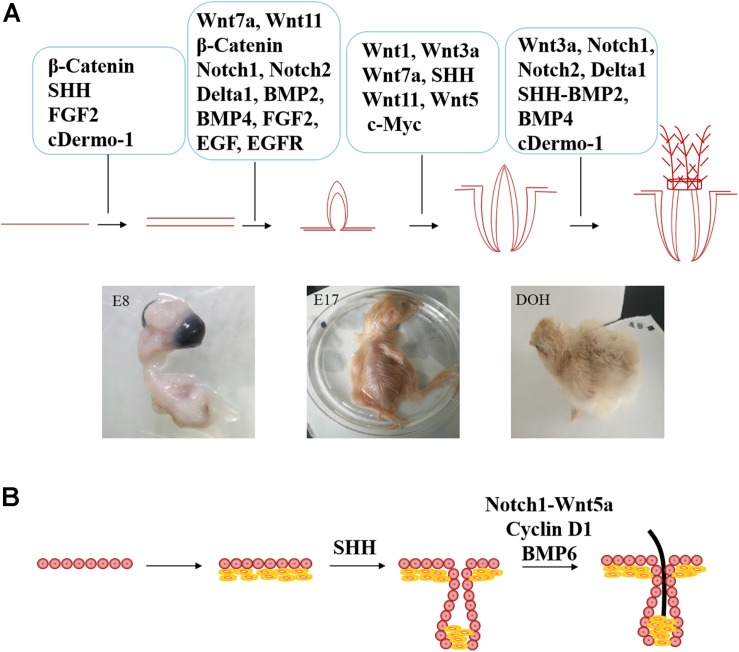
Comparison of different signaling molecules involved in the regulation of feather follicle and hair follicle development. **(A)** The molecular signaling that involved in poultry feather follicle development (E8 = day 8 of incubation, E17 = day 17 of incubation, and DOH = day of hatching. **(B)** Extra signaling molecules involved in hair development.

Feather development regulation starts from the changes in the adjacent microenvironment sensed by the basal filamentous pseudopods, which dynamically regulate the proliferation and differentiation of feather follicle stem cells, thereby affecting the formation of feather follicles and the process of feather bifurcation. Previous studies have found that signaling pathways such as Wnt, SHH, Notch and BMP, including their ligands, receptors and signaling molecules, regulate the development and cycle of feather follicles (Figures 3, 4A).

### Wnt Signaling

Wnt signaling regulates feather follicle morphogenesis and feather growth by regulating the development of the dermis, feather bundles and feather buds (Lin et al., 2006). Wnts trigger three downstream signaling pathways: the classical Wnt/β-catenin signaling pathway and non-canonical signaling pathways (Wnt/Ca^2+^ pathway and planar cell polarity (PCP) pathway). The Wnt/β-catenin signaling pathway plays a key role in the regulation of feather follicle morphogenesis and skin remodeling.

#### Wnt/β-Catenin Signaling Pathway

TH3he classical Wnt pathway mainly includes the Wnt signaling protein, the membrane receptor FZD family, cytosolic β-catenin and the nuclear LEF/TCF transcription factor family. Wnt ligands bind to the Frizzled receptor via the low-density lipoprotein receptor LRP5/6 and transmit signals to Dsh. Activated Dsh reduces the activity of degradation complexes composed of APC, Axin, GSK-3β and PP2A; inhibits the degradation of β-catenin (Giles et al., 2003; Saito-Diaz et al., 2013); and promotes its accumulation in the cytoplasm, as well as transfer to the nucleus. Finally, β-catenin binds to TCF/LEF1 to replace the transcriptional suppressor Groucho in the target gene promoter, thereby regulating the expression of downstream target genes (c-Myc, Cyclin D1, etc.). This pathway activates both the proliferation and differentiation of feather follicle stem cells (Lin et al., 2006).

Wnt ligands are necessary for feather follicle morphogenesis and feather growth. Wnt7a and Wnt11 are related to feather follicle initiation, in which Wnt7a is involved in the location of feather buds (Widelitz et al., 1999), and Wnt11 helps to determine the boundary of feather buds (Chang et al., 2004) by regulating the interbud domain. Moreover, Wnt7a can elongate feather buds to promote the development of feather follicles (Widelitz et al., 1999). Chang et al. (2004) reported that Wnt1 and Wnt3a activated the classical Wnt signaling pathway and positively affected the formation of feathers. When the positive regulator is dominant, the bud is unusually thick. Similarly, the activation of c-Myc, a protein downstream of Wnt, also resulted in increased feather buds (Chiu, 2008). This result suggests that Wnt plays a positive role in the development of feather follicles, possibly by regulating the expression of downstream c-Myc. Studies on chicken feathers have found that inhibition of Wnt3a transforms bilaterally symmetric feathers (contour feathers) into radially symmetric feathers (downy feathers). Wnt3a may also play an important role in feather branching (Yue et al., 2006).

Wnt ligands can regulate the activity of β-catenin, which is the central link of the Wnt/β-catenin signaling pathway. In the early stage, β-catenin is involved in the formation of the track. Subsequently, β-catenin and Wnts together regulate the entire feather follicle structure and the interbud domain (Noramly et al., 1999; Widelitz et al., 2000), and increased β-catenin activity promotes better feather follicle growth (Widelitz et al., 2000). Furthermore, the absence of β-catenin in hair leads to hair follicle development stagnation and a decrease in the number of hair follicles (Lin et al., 2006), but this phenomenon needs to be further verified in feather follicles.

Cyclin D1 is a downstream target gene of β-catenin that can regulate the proliferation and differentiation of hair follicle stem cells. Therefore, as shown in Figure 4B, Cyclin D1 controls hair follicle development by regulating the proliferative activity of hair follicle stem cells and transiently amplifying cells (Mill et al., 2003). However, this phenomenon need to be further verified in feather follicles.

#### Non-canonical Signaling Pathway

Similar to the canonical Wnt signaling pathway, non-canonical Wnt signaling pathways, including the Wnt/Ca^2+^ and PCP signaling pathways, require Wnt proteins to bind to a cysteine-rich domain at the amino terminus of the Frizzled receptor on the cell membrane but will not cause β-catenin accumulation. The Wnt/Ca^2+^ pathway is mainly activated by Wnt5, which promotes the production of calcium ions by phospholipase C (PLC) and further acts on protein kinase C (PKC) and calmodulin-dependent protein kinase II (CAMKII). PKC and CAMKII affect gene transcription by dephosphorylation of the nuclear factor of activated T cells (NF-AT) (Kühl et al., 2000).

PCP genes have been identified *Drosophila* as being important for establishing polarity in various processes, including feather follicle orientation. During the formation of chicken embryonic feather buds, PCP genes are potentially involved in polarity (Chiu, 2008; Lin and Yue, 2018). To date, few studies have focused on exploring the mechanism by which the non-classical PCP pathway regulates follicle morphogenesis. In general, in PCP pathways, Wnt11 activates disheveled associated activator of morphogenesis-1 (DAAM1) and protein kinase B (PKB) through Dvl in the cytoplasm, while DAAM1 positively regulates Rho-associated protein kinase 2 (ROCK2) to affect cytoskeleton formation, and PKB activates c-Jun N-terminal kinase (JNK). These regulatory proteins affect the transcription of multiple genes. A previous study also found that Wnt11 can increase the interbud domain (Chang et al., 2004), but whether it works through only the PCP pathway needs to be clarified. Similarly, Wnt5 and Wnt11 negatively affect the development of poultry feather follicles through non-canonical Wnt signaling pathways (Chang et al., 2004). When the negative regulatory wnts dominates, the feather buds lengthen more rapidly, and the diameter of the feather was reduced (Chang et al., 2004).

The ligands of the Wnt signaling pathway and their key proteins play a positive or negative regulatory role in the development of feather follicles and feather growth in poultry. However, the specific mechanism of the Wnt signaling pathway needs to be further studied, and research on mammalian hair may provide a good reference for future work.

### SHH Signaling Pathway

Sonic Hedgehog (SHH), a member of the Hedgehog (Hh) signal protein family, is a necessary signal transduction pathway for feather follicle development. It mainly participates in mitosis and morphogenesis during dermal papilla maturation and feather bud development (McKinnell et al., 2004). SHH is an important factor for controlling the transition from the telogen to the growth stage of feather follicles.

The SHH signaling pathway is highly conserved in evolution, and its components include ligands [patched (ptc) and smo], Gli family members and downstream targets. Mechanically, the SHH precursor is activated by acyltransferase and then binds to the receptor Ptc on the cell membrane, dissociates the Ptc-Smo complex and releases Smo, thereby disrupting the inhibitory effect of Ptc on Smo activity. When free Smo enters the cytoplasm, it activates downstream Gli family zinc finger transcription factor to complex with protein kinase A (PKA), which moves into the nucleus and activates the transcription of downstream target genes (Cohen, 2003).

SHH is mainly expressed in the epidermis of feather follicles during feather development and mediates the key interaction between epithelial and mesenchymal cells (Nohno et al., 1995; Ting-Berreth and Chuong, 1996). When SHH was inhibited, feather buds became irregular and fused (El-Magd et al., 2014). Overexpression of exogenous SHH during feather development expanded feather bud formation (Ting-Berreth and Chuong, 1996). Li et al. (2018) found that in the normal process of chicken feather elongation, SHH-responsive mesenchymal cells displayed synchronized Ca^2+^ oscillations, and inhibition of the SHH signal changed the mesenchymal Ca^2+^ distribution and feather elongation. SHH and Wnt/β-catenin were shown to coactivate the expression of Connexin-43, establish a gap junction network, synchronize the distribution of Ca^2+^ among cells and coordinate the cell movement mode (Li et al., 2018).

Studies have shown that the downregulation of SHH expression inhibits dermal papilla cell condensation and maturation, resulting in inhibition of hair follicle formation, as shown in Figure 4B (Chiang et al., 1999). Knocking out the transcription factor SOX9 gene downstream of the SHH signaling pathway will reduce epidermal regeneration (Nowak et al., 2008). However, exogenous SHH can increase the expression of Gli, activate dermal papilla cells and improve the ability of hair follicle formation (Lee and Tumbar, 2012). Whether SHH can activate the growth of feather follicle dermal papilla cells needs further verification.

### Notch Signaling Pathway

Notch signaling can promote or inhibit cell proliferation, cell death, the acquisition of specific cell fates, and the activation of differentiation processes. These processes occur in cells throughout the entire process of organism development and in adult tissues that maintain self-renewal. The release of intracellular notch fragments depends on the proteolytic cleaveage of receptor proteins after ligand binding. After its release by proteolysis from a membrane tether, the Notch intracellular domain (NICD) translocates to the nucleus. There, the NICD associates with a DNA binding protein to assemble a transcription complex that activates downstream target genes (Kopan and Ilagan, 2009). Importantly, Notch/Delta signaling plays a role in early feather pattern formation and feather growth.

A previous study reported that Notch 1 and Notch 2 mRNAs are expressed in the skin before the initiation of feather buds in a localized pattern. In the early stages of feather bud development, the ligand Delta 1 and Notch 1 are localized to the forming buds, while the expression of Notch 2 is excluded from the bud. Delta1 is expressed in the dermis, whereas Notch 1 expression is restricted in the epithelial placode. Therefore, the complementary expression of Delta in the dermis with Notch 1 in the epidermis suggests that this signaling promotes feather growth. In contrast, Notch 2 transcripts have been observed in the dermis adjacent to each shoot, indicating that Notch 2 activity inhibits feather growth (Crowe et al., 1998). During the branching of the feather, Notch 1 is enriched in the prefeathered epithelium and is expressed in basal keratinocytes at low levels. After branching, Notch 1 is enriched in the barb plate (Cheng et al., 2018). Thus, Notch and Delta 1 are expressed at the correct time and place to participate in the formation of the feather pattern. Once the initial buds form, the expression of Notch and its ligands is observed within each bud, whereas Delta 1 transcripts are downregulated. These results indicate that Notch and Delta are involved in the formation of the feather array, and Delta 1 exerts the following two effects in the early stage of feather formation: promoting feather growth by activating Notch 1 and inhibiting feather growth by activating Notch 2 (Crowe et al., 1998).

Previous studies have shown that Notch and Wnt signaling pathways interact to regulate hair follicle growth. Notch1 can activate Wnt5a expression (Hayward et al., 2005; Proweller et al., 2006). As shown in Figure 4B, Wnt5a regulates hair follicle differentiation by promoting Foxn1 gene expression (Mecklenburg et al., 2001), but its role in feather follicle development remains uncertain.

### BMP Signaling Pathway

Bone morphogenetic proteins (BMPs) belong to the TGFβ superfamily of ligands and play an important role in the development of feather follicles and feathers. BMP-induced signal transduction by the extracellular BMP ligand involves binding to the BMP receptor complex on the cell membrane, which allows the type II receptor to activate the type I receptor by phosphorylation. The activated type I receptor phosphorylates the serine residue at the R-Smad end of the regulatory receptor and binds to a Co-Smad to enter the nucleus and regulate the transcription of the target gene under the action of different DNA binding proteins.

In the process of feather follicle development, BMP mainly plays an inhibitory role (Jung et al., 1998). Drm/Gremlin inhibits BMP and limits the inhibitory effect of BMPs, allowing the adjacent row of feathers to form (Bardot et al., 2004). However, the combination of BMP with other factors can relieve this inhibition to balance these proteins and thus regulate the growth of feather follicles and feathers. The derived SHH-BMP2 signaling pattern is related to the development of feather structure. The longitudinal SHH-BMP2 expression domain in the marginal plate epithelium between the barb ridges provides the anterior form of barbs and rachis. Therefore, SHH-BMP2 may be involved in the feather branching morphology (Harris et al., 2002). It was also confirmed that antagonizing BMP4 with Noggin (a BMP signal antagonist) controls feather branching (Yu et al., 2002). BMP4 promotes the formation of the rachise, while Noggin promotes the formation of barb ridges. In addition, the combination of Noggin and sonic hedgehog (SHH) has been shown to induce feathered skin (Fliniaux et al., 2004).

Kobielak et al. (2007) found that BMP was stably expressed in the microenvironment of hair follicle stem cells, and knockout of the BMP receptor could lead to overactivation of hair follicle stem cells. BMP6 can inhibit the proliferation of hair follicle cells and the growth of hair follicles by maintaining the resting state of stem cells (Clavel et al., 2012). However, this process requires further verification in feather follicles.

### Other Signaling Pathways

Many members of the FGF family are involved in the regulation of feather follicle development. For example, FGF2 can induce the formation of dense dermal tissue in wild-type chickens, regulating the normal growth of feathers. The FGF2 can induce the formation of numerous feather buds (Song et al., 2004). Studies have shown that cDermo-1 leads to the formation of dense dermal tissue because of its overexpression and induces continued feather growth. In contrast, EGFR inhibitors shorten the distance between buds and increase the number of feather buds. The EGF signal acts directly on the epidermis and functions independently of BMP signaling (Atit et al., 2003).

## Nutrition and Feather Growth

Feather follicles drive structural renewal through feather follicle stem cell proliferation and differentiation (Yue et al., 2005). Importantly, feather follicle tissue is highly malleable, and dietary deficiencies can contribute to the obvious atrophy of the feather follicle, malformed feathers, fragile feathers, and feather loss. Dietary nutrition, especially crude proteins, amino acids, minerals, and vitamins, plays a key role in regulating the development of feather follicles and the growth of feathers (Supplee, 1966; Taylor, 1967; Urdaneta-Rincon and Leeson, 2004; Zeng et al., 2015). Therefore, it is important to dissect the effects of dietary nutrients on feather morphogenesis and the molecular mechanisms of feather follicle homeostasis. However, the effects of these nutrients on signal transduction in feather follicle cells are extremely complex. In the past decade, much has been learned about potential nutritional influences on feather growth and molting, yet little is known about how nutrients affect the signaling that regulates feather growth. The effects of related nutrients on feather growth are summarized in Table 1.

**TABLE 1:** Effect of dietary supplementation of various nutrients on poultry feathers.

**Animal**	**Time**	**Nutrient**	**Dose in the diet**	**Influences**	**References**
Male Ross broiler chicken	0–3 weeks of age	Crude protein (CP)	17, 21, 25, 29%	Feather is the heaviest with a diet containing 25% CP	Urdaneta-Rincon and Leeson, 2004
Ross 308 broiler chicken	2–22 weeks of age	CP	12–13%, 14–16%	High-protein diet group has higher feather coverage	Van Emous et al., 2015
Ross 308 broiler chicken		Methionine	Injected into the yolk: 20, 30, 40, 50 mg	Density and diameter of feather follicles are increased significantly in the presence of 50 mg of methionine	Nazem et al., 2015
Peking ducklings	15–35 days of age	Methionine	0.3, 0.39, 0.45, 0.56, 0.68%	Compared with that in the 0.45 and 0.56% methionine supplementation groups, feather coverage is increased significantly	Zeng et al., 2015
Male broiler chicken	0–3 weeks of age	Valine	0.63, 0.83%	When valine is supplemented (0.83%), feather abnormalities are repaired	Farran and Thomas, 1992
Cornish-crossbreed chicken	8–17 days of age	Valine	0.60, 0.68, 0.76, 0.82%	The rough and curved appearances of the feathers gradually increase as the proline level in the diet decreases	Robel, 1977
Cornish-crossbreed chicken	8–17 days of age	Leucine	0.60, 0.68, 0.76, 0.82%	The rough and curved appearances of the feathers gradually increase as the level of leucine in the diet decreases	Robel, 1977
Cornish-crossbreed chicken	8–17 days of age	Isoleucine	0.32, 0.38, 0.44, 0.50%	The rough and curved appearances of the feathers gradually increase as the level of isoleucine in the diet decreases	Robel, 1977
Female ring-neck pheasant chicken	0–3 weeks of age	Zn	Basal diet + 60 mg/kg, basal diet + 120 mg/kg	Adding 60 and 120 mg/kg to the basal diet can effectively reduce feather fraying	Cook et al., 1984
Leghorn female chicken	0–3 or 4 weeks of age	Zn	52, 78, 156, 208 mg/kg	When diets containing 78, 156, and 208 mg/kg zinc are fed for 1 week or 156 mg/kg zinc are fed for three weeks, feather fraying is almost zero	Sunde, 1972
Male broiler chicken	0–6 weeks of age	Zn	4.4, 8.4, 10.4%	Under high-temperature conditions (30, 28, and 26°C), broilers fed a 4.4% Zn ration had significantly higher feather phosphorus levels than those fed other rations	Lai et al., 2010
Ring-necked pheasant chicken	Not given	Zn	42, 47, 52, 62 mg/kg	Adding 62 mg/kg zinc produces satisfactory feathering	Scott et al., 1959
Female crossbred chicken (New Hampshire male × Columbian female)	0–27 days of age	Sn, V, Cr, Ni	2, 1, 3, 3 mg/kg	No influence	Baker and Molitoris, 1975
Broiler chicken	0–42 days of age	Organic selenium yeast	0.1, 0.3 mg/kg	Organic Se improves the feathering rate	Edens et al., 2001
Ring-necked pheasant	Not given	Niacin	22, 33, 44, 55, 66, 77 mg/kg	Adding 55 mg/kg niacin or more produces satisfactory feathering	Scott et al., 1959
Chicken	3–6 weeks of age	B group vitamins	Deficient	Abnormal flight feathers	Taylor, 1967
Turkey	0–26 days of age	Vitamin E and selenium	Deficient	Abnormal flight feathers	Supplee, 1966

### Protein

The protein content of poultry feathers is as high as 89–97%. Therefore, the dietary crude protein level is considered the main nutrient factor affecting feather growth and development (Fisher et al., 1981). Urdaneta-Rincon and Leeson (2004) found that the feather weight increased with dietary crude protein levels when the dietary crude protein content was between 17 and 25% in Rose broilers. Similarly, a low dietary crude protein feeding (12–13% vs. 14–16%) during rearing and the first lay phase can lead to poor feather cover in broiler breeder females (Van Emous et al., 2015). The feathers are mainly composed of corneous materials made of α-keratins and β-keratins. In addition, α-keratin and β-keratin networks are interdependent, and mutations of either type of keratin will inhibit the formation of appropriate barbs (Wu et al., 2015). A previous study found that the expression of a mutant α-keratin gene *KRT75* leads to frizzled feathers in domestic chickens (Ng et al., 2012).

### Amino Acids

Approximately 88–90% of feathers are composed of the protein keratin, which requires a high level of sulfur-containing amino acids cystine and methionine for its production (Wheeler and Latshaw, 1981). While some of these amino acids are produced by various tissues in the body, others must be supplemented by diet. Cystine is the main component of keratin, while methionine is converted into cystine by transsulfuration in the feather follicle and liver (Xu et al., 2010). A previous study has shown that *in ovo* injection of methionine can contribute to feather follicle development and feather growth, which is characterized by primary and secondary feather follicles (Nazem et al., 2015). Within a certain range, the quality of feathers is directly proportional to the level of methionine. Zeng et al. (2015) found that compared with that in the 0.45 and 0.56% methionine supplementation groups, the feather coverage of 35 days old ducks in the 0.30 and 0.39% methionine supplementation groups was significantly decreased, and the most suitable dietary methionine supplemental level was 0.484%. These results illustrated that sulfur-containing amino acids, which have been considered the first-limiting amino acids in most practical diets for poultry, play an important role in feather follicle development and feather growth. However, current research on the promotion of feather follicles by methionine is limited to descriptions of its impact of growth and the role in feather follicle stem cells has yet to be reported. Studies have found that methionine deficiency inhibits the proliferation of intestinal stem cells (Saito et al., 2017). After methionine supplementation, the ability of intestinal stem cells to divide is enhanced via the primary metabolite S-adenosylmethionine (SAM), which promotes protein synthesis in stem cells (Obata et al., 2018). Lauren et al. (2019) showed that the lack of methionine inhibited the Wnt/β-catenin signaling pathway and that Wnt/β-catenin was reactivated after the addition of SAM. The precise regulation of protein synthesis is equally important for the proliferation and maintenance of hair follicle stem cells (Blanco et al., 2016). However, the metabolism of methionine in feather follicle stem cells and how the Wnt signaling pathway mediates this metabolic process and then regulates the mechanism of feather growth remain unclear. In addition, SAM, an important methyl donor, is involved in regulating the distribution and translocation of various proteins. However, whether it relies on methylation to control the translation process and thus regulate feather follicle stem cell activity needs to be further verified.

Apart from studies with sulfur-containing amino acids, the metabolism of branched-chain amino acids has an important influence on the development of feather growth. Branched-chain amino acids (valine, isoleucine, and leucine) are relatively abundant in feather proteins. Reduced valine in the diet (0.63%) decreased the protein content in the broiler feathers and caused abnormal feathers. When valine was supplemented (0.83%), the protein content in the feathers increased, and feather abnormalities were not observed (Farran and Thomas, 1992). Feather abnormalities were observed in chicks lacking valine, leucine and isoleucine. As the levels of valine, isoleucine and leucine in the diet decrease, the rough and curved appearance of the feathers gradually increases, and diets containing 0.70% valine result in optimum feather growth (Robel, 1977).

### Minerals

Lack of mineral elements in the diet can also affect feather growth, and zinc levels have been the most extensively studied. Cook et al. (1984) have shown that adding 60 or 120 mg/kg zinc to a commercial mixed diet can effectively reduce the fraying of chicken feathers. In another study, the addition of zinc was effective at reducing the incidence of chicken feather abnormalities. When the total zinc level in the diet was dropped to 52 mg/kg, fraying occurred. However, when the total Zn level in the diet was increased to 78 mg/kg or higher, feather fraying was effectively reduced after only the first week of feeding. Feeding high levels of zinc for only the first week of life provided excellent protection from feather fraying (Sunde, 1972). Lai et al. (2010) found that at high temperatures, the feather defects of broiler chicks with Zn contents of 84 and 104 mg/kg were lower than the dietary Zn content of 44 mg/kg. However, at lower ambient temperatures, there was no significant difference in the feather coverage between the high- and low-Zn groups. Meaningfully, this finding shows that Zn can alleviate feather growth defects under high-temperature conditions. Notably, downregulation of the Wnt/β-catenin signaling pathway is related to the proliferation deficit induced by zinc deficiency in neural stem cells, and application of lithium chloride (LiCl, GSK-3β inhibitor) was shown to reverse the impairment of cell proliferation via upregulating β-catenin (Zhao et al., 2015). However, it is not clear whether zinc promotes the development of feather follicles and feathers by regulating Wnt/β-catenin signal activity.

Additionally, Supplee (1966) found that a lack of selenium in the diet affects the normal growth and development of feathers. Supplementation with organic selenium can effectively improve the feathers of broiler chickens (Edens et al., 2001). Other studies found that the addition of 2 mg/kg tin, 1 mg/kg vanadium, 3 mg/kg chromium and 3 mg/kg nickel did not influence feather growth over a 27 days assay period (Baker and Molitoris, 1975). In summary, although mineral contents may be low, they play an important role in the growth and development of feathers in poultry.

### Vitamins

Vitamins, as coenzymes are required for normal feather development and growth in poultry. In a 10 years study, Taylor observed that the lack of B vitamins (pantothenic acid, folic acid, biotin, and niacin) in the diet caused abnormalities in the feathers of chickens from 3 to 6 weeks of age (Taylor, 1967). Moreover, Scott et al. (1959) found a dietary niacin content of 55 mg/kg produced satisfactory feathers. Another study observed unusual feather development when chicks received diets deficient in vitamin E (Supplee, 1966). Therefore, the appropriate vitamin level is essential for the growth and morphological maintenance of feathers.

Among the vitamins the role of vitamin D3 is most well established for its influence on hair follicle development and the hair cycle. Vitamin D is involved in regulating cell proliferation, differentiation and apoptosis as well as in promoting hair follicle regeneration (Vijaya et al., 2002). The vitamin D derivative calcipotriol increases the proportion of cells in the hair follicle growth phase/rest phase (Amor et al., 2010), while vitamin D receptor knockout leads to hair loss and increased cell numbers at the end of the growth phase and the degenerative phase, decreased keratinocyte proliferation activity, and hair follicle growth blockage. This result suggests that vitamin D initiates the hair follicle growth cycle (Luderer and Demay, 2010). Additionally, the loss of vitamin D receptors leads to a decrease in Lef1 at the end of the degenerative phase, leading to its inability to bind to β-catenin and resulting in atrophy of hair follicle development (Bikle, 2010). During this process, SHH, Gli1, and Gli2 expression levels are decreased, and the hair follicle growth cycle is disordered. Therefore, vitamin D may initiate the hair follicle cycle through the vitamin D receptor, thereby regulating the growth and development of hair follicles. Vitamin D is a crucial supplement in poultry feed necessary for healthy bone development and further investigations of its role of in plumage quality may add to further understanding of this vitamin/hormone signaling system in appendage growth.

As mentioned above, various nutrients were previously studied separately to assess their effects on feather growth. However, it is worth noting that cooperative effects may exist between several nutrients, and the overall mechanism underlying how nutrients regulate feather growth remains unknown. Thus, exploring the molecular mechanism underlying the interaction between nutrients and feathers will be meaningful for promoting the growth and development of poultry feathers.

## Conclusion

Feather follicle stem cell-driven development and regeneration are dependent on the regulation of different signals (e.g., Wnt, SHH, Notch, and BMP). These signals integrate to form a fine and dense gene network system, regulate the fate of stem cells in an orderly fashion, and interact in dermal and epidermal cells. The feather follicles are formed underneath and eventually bifurcate to form a complete feather structure. Nutrients are not only the material basis for feather follicle and feather development but also serve as mediators triggering signal transduction networks in the feather follicle stem cell microenvironment. Their deficiencies generally lead to severe feather loss or structural abnormalities that reduce the profits of rearing poultry. However, the intricate linkages among nutrient-mediated feather follicle development, regeneration processes and signaling pathways through various signaling molecules are unclear. Therefore, it is necessary to further understand the mechanism of action of nutrients upon the feather follicle stem cell microenvironment, to provide a theoretical basis for novel interventions that can enhance plumage coverage, during critical periods of the commercial poultry lifespan.

## Author Contributions

MJC and CQG conceived the ideas. MJC, WYX, SGJ, XQW, and HCY performed the literature search and contributed to the writing of the manuscript.

## Conflict of Interest

The authors declare that the research was conducted in the absence of any commercial or financial relationships that could be construed as a potential conflict of interest.
